# GRMD cardiac and skeletal muscle metabolism gene profiles are distinct

**DOI:** 10.1186/s12920-017-0257-2

**Published:** 2017-04-08

**Authors:** Larry W. Markham, Candice L. Brinkmeyer-Langford, Jonathan H. Soslow, Manisha Gupte, Douglas B. Sawyer, Joe N. Kornegay, Cristi L. Galindo

**Affiliations:** 1 Department of Medicine, Division of Cardiovascular Medicine, Vanderbilt University Medical Center, 2200 Pierce Avenue, 359A Preston Research Building, Nashville, TN 37232 USA; 2 Department of Veterinary Integrative Biosciences, Texas A&M University, College Station, TX USA; 3 Department of Pediatrics, Division of Pediatric Cardiology, Vanderbilt University Medical Center, Nashville, USA

**Keywords:** Duchenne, BDNF, Muscular dystrophy, Cardiac, Dystrophin, Metabolism

## Abstract

**Background:**

Duchenne muscular dystrophy (DMD) is caused by mutations in the *DMD* gene, which codes for the dystrophin protein. While progress has been made in defining the molecular basis and pathogenesis of DMD, major gaps remain in understanding mechanisms that contribute to the marked delay in cardiac compared to skeletal muscle dysfunction.

**Methods:**

To address this question, we analyzed cardiac and skeletal muscle tissue microarrays from golden retriever muscular dystrophy (GRMD) dogs, a genetically and clinically homologous model for DMD. A total of 15 dogs, 3 each GRMD and controls at 6 and 12 months plus 3 older (47–93 months) GRMD dogs, were assessed.

**Results:**

GRMD dogs exhibited tissue- and age-specific transcriptional profiles and enriched functions in skeletal but not cardiac muscle, consistent with a “metabolic crisis” seen with DMD microarray studies. Most notably, dozens of energy production-associated molecules, including all of the TCA cycle enzymes and multiple electron transport components, were down regulated. Glycolytic and glycolysis shunt pathway-associated enzymes, such as those of the anabolic pentose phosphate pathway, were also altered, in keeping with gene expression in other forms of muscle atrophy. On the other hand, GRMD cardiac muscle genes were enriched in nucleotide metabolism and pathways that are critical for neuromuscular junction maintenance, synaptic function and conduction.

**Conclusions:**

These findings suggest differential metabolic dysfunction may contribute to distinct pathological phenotypes in skeletal and cardiac muscle.

**Electronic supplementary material:**

The online version of this article (doi:10.1186/s12920-017-0257-2) contains supplementary material, which is available to authorized users.

## Background

Duchenne muscular dystrophy (DMD) and the genetically homologous *mdx* mouse and golden retriever muscular dystrophy (GRMD) dog models are caused by mutations in the *DMD* gene, resulting in severely reduced or absent dystrophin protein [1–5]. Despite being genetically homologous, the three diseases demonstrate distinct phenotypes, with DMD and GRMD being more severe [4, 6]. Gene expression in *mdx* mice and DMD patients is also distinctive. By 16 weeks of age, the *mdx* transcriptome is relatively quiescent [7], while DMD expression profiles demonstrate a so-called “metabolic crisis” [8, 9]. Meta-analysis of gene expression datasets from six different studies of DMD skeletal muscle biopsies provide especially strong evidence that thematic metabolic disturbances are pathophysiologically relevant [10] and likely contribute to muscle atrophy [11]. In support of this concept, therapies that enhance metabolism, such as corticosteroids and coenzyme Q, are temporarily beneficial.

In contrast to the early progressive wasting seen with DMD skeletal muscle, the heart is relatively preserved, with onset of cardiomyopathy typically occurring in the second or third decade of life [12, 13]. How or why the heart is temporarily spared is not understood, and predictive markers for DMD cardiomyopathy are currently unavailable. As with skeletal muscle, the mdx and GRMD models are distinctive. As with DMD, GRMD dogs have progressive skeletal muscle weakness and late-onset cardiomyopathy [5, 14–16], while the *mdx* mouse exhibits subtle cardiac abnormalities [6]. We hypothesized that differences in cardiac and skeletal muscle metabolism contribute to this variable disease progression. To address this hypothesis, we studied a group of GRMD dogs that were previously shown to have altered expression of osteopontin (OPN) and brain-derived neurotropic factor (BDNF) in dystrophic skeletal and cardiac muscles, respectively [17]. These findings were subsequently translated to boys with DMD, demonstrating that circulating levels of OPN and BDNF correlated with skeletal muscle function or cardiac dysfunction [17]. Building on this work in the current study, we found strikingly different metabolic gene expression patterns in cardiac and skeletal tissue, providing further insight into potential molecular mechanisms underlying tissue-specific disease progression.

## Methods

### Animals

The study was approved by the Institutional Animal Care and Use Committee at the University of North Carolina at Chapel Hill. All dogs were used and cared for according to principles outlined in the National Research Council Guide for the Care and Use of Laboratory Animals. The GRMD genotype was suspected based on elevated serum creatine kinase and confirmed by genotyping. Affected dogs subsequently developed characteristic clinical signs. Notably, the GRMD phenotype progresses dramatically over the 3 to 6 month age period and then tends to stabilize [18, 19]. Given the relative equivalency of the first year of a golden retriever’s life to the initial 20 years for a human [20], the 3 to 6 month period for GRMD corresponds to an analogous period of deterioration between ages 5 and 10 years in DMD [21–23]. A total of 15 dogs were included in the study: 3 each 6–7.5 and 12–13 month-old GRMD dogs and 6 age-matched (littermate) wild type controls, plus 3 older (47, 52, and 93 month-old) GRMD dogs. Samples from the medial head of the gastrocnemius (MHG) and left ventricular (LV) free wall were removed at necropsy and processed. Muscle sections were snap frozen in a Freon substitute, cooled in liquid nitrogen, stored at -80 °C and then shipped in cryovials to Vanderbilt for analysis.

### Microarrays

Total RNA was isolated from the LV and MHG of the 15 dogs according to the manufacturer’s protocol (Qiagen, Germantown, MD). Quality assessment of RNA, further processing, and data acquisition were performed by the GSR Microarray Core at Vanderbilt. Affymetrix Canine Gene 1.0 Expression arrays (Affymetrix, Inc., Santa Clara, CA) were used, two arrays per animal (for paired LV and skeletal muscle samples), for a total of 30 arrays. Raw data were RMA normalized, followed by ANOVA with Benjamini & Hochberg correction, using Partek Genomics Suite 6.6 (Partek Incorporated, St. Louis, MO). Genes with a *p* value (with or without FDR) < 0.05 and fold-difference > 1.5 were considered significantly altered. Normalized and raw data generated from canine gene expression arrays were deposited under Accession Number GSE68626 in the Gene Expression Omnibus (GEO), available through NCBI (http://www.ncbi.nlm.nih.gov/geo/)GEO. Functional and pathway enrichment analyses were conducted using Ingenuity Pathway Analysis (IPA, Qiagen, Germantown, MD), Partek Genomics Suite 6.6 (Partek Incorporated, St. Louis, MO), DAVID Bioinformatics Resources 6.8 [24, 25],or Gene Set Enrichment Analysis (GSEA) desktop freeware version 2.2.3 (BROAD Institute). For GSEA using Partek software, normalized signal values were used. For GSEA using BROAD software, differentially expressed genes were first separated by directionality (up-regulated genes and down-regulated gene) and the subsequent lists ranked by *p* value, where ranked score = -log(*p* value).

### Western blot analysis

Western blot analysis was performed as previously described [26]. Primary antibodies used were rabbit anti-AMPK (total and phosphorylated, Cell Signaling Technologies, Inc., Danvers, MA) and rabbit anti-p90 (Cell Signaling Technologies) as a loading control.

### Statistics

All data are expressed as means ± SEM. Comparisons made between two variables were performed using a Student *t* test unless otherwise stated. *P* values of less than 0.05 were considered statistically significant. For gene microarray analysis, data were uploaded into Partek Genomics Suite version 6.6 (Partek Incorporated, St. Louis, MO) and RMA normalized before further analysis. Partek was used to perform pairwise comparisons of average group values and one-way ANOVA for the eight groups. Only probes that resulted in a fold-change of at least 1.5 and *p* value of less than 0.05 were considered significantly altered.

## Results

### GRMD gene expression findings: an overview

**Fig. 1:**
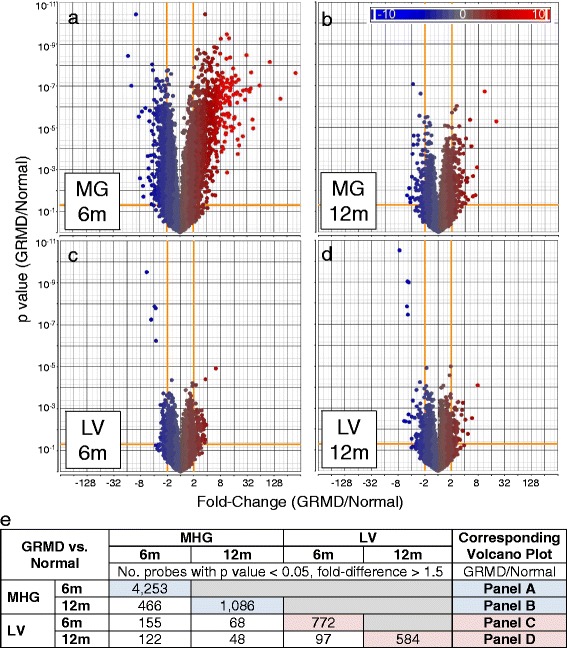
Volcano plots are shown highlighting gene expression differences between dystrophic medial head of the gastrocnemius (MHG) skeletal muscle of golden retriever muscular dystrophy (GRMD) dogs at 6 (**a**) and 12 (**b**) months (m), as well as GRMD *left ventricle* (LV) from 6 (**c**) and 12 (**d**) month-old dogs, each compared to their respective wild type controls. The y-axis displays *p* values, and fold-differences are plotted on the x-axis for GRMD versus control dogs for each of the four comparisons. Each circle represents an individual probe set (gene) and is colored based on fold-difference (*blue* for genes with *lower* expression in GRMD dogs, red for genes with higher expression values, and *gray* for no change), as shown in the legend. *Orange lines* mark significance cut-off values (*p* value < 0.05, fold-difference > 1.5). Panel (**e**) shows a matrix including the number (No.) of array signal intensity probes deemed as significantly differential (*p* value < 0.05, fold-difference > 1.5) between pairs of groups. Pairwise comparisons include GRMD versus wild-type (WT) dogs for matched skeletal muscle (MHG) and LV tissue of dogs aged 6 months and 12 months. Pairwise comparisons of the two age groups for each tissue are also shown

A total of 30 samples from 15 dogs were included in the study, divided according to disease-state (GRMD or wild type) and tissue type (skeletal [MHG] muscle or cardiac [LV] muscle). We focused primarily on 12 dogs at ~6 months or 12 months, for which there were a total of 8 groups with 3 biological replicates each. Based on a non-stringent and straightforward analysis of age-matched and wild type samples, we compiled a single list of 5,835 probes that were significantly differential (*p* value < 0.05, fold-difference > 1.5) between GRMD and wild type tissues for at least one of the four comparisons (Additional file 1: Table S1). An overview of statistical results is shown in Fig. 1e. The most immediately obvious distinction among the tissues was the overall difference in gene expression profiles between the 6 and 12-month GRMD age groups. Whereas there were relatively few expression differences between LV samples at these two ages when compared to wild type control animals, MHG samples from these same dogs exhibited dramatic differences (Fig. 1). This is consistent with the more dramatic progression of skeletal muscle disease up to 6 months of age with subsequent stabilization, while cardiac muscle function is typically normal over this period. Hierarchical clustering of all 5,835 probes also indicated that the global transcriptional profile of GRMD MHG at 6 months was unique and distinct from all other samples, including GRMD MHG at 12 months (Additional file 2: Figure S1).

Similar to what has been reported elsewhere for DMD and animal models [27–29], up-regulated skeletal muscle genes included those associated with fibrosis (e.g., collagens, *POSTN*, *TIMP1*), inflammation (e.g., complement components, chemokines, cytokines), and muscle cytoskeletal proteins (e.g., myosin heavy chains, troponins, and actins). The most profoundly down-regulated genes were those associated with muscle growth and development (e.g., *CLCN1*, *MYLK2*, *MYBPC2*, and *MSTN*). There were surprisingly few genes (9.6%) that were differentially expressed between GRMD and wild type MHG tissue in both of the ages evaluated. The majority of these genes were altered more profoundly in the younger GRMD dogs relative to wild type controls. Embryonic and perinatal forms of myosin heavy chains (*MYH3* and *MYH8*) for instance were up-regulated 180-fold and 61-fold in MHG at 6 months, compared to 6-fold and 7.4-fold in 12-month-old dogs, respectively. Up-regulated genes with a higher magnitude of increase in MHG from 12- versus 6- month-old GRMD dogs included those encoding adult myosin heavy chains, extracellular matrix components, parvalbumin, and β-1-syntrophin (*SNTB1*).

Cardiac expression differences included genes with disease-associated functions, such as muscular disorders, cardiovascular disease, and organismal injury. To identify non-directional (up or down) and non-age related (i.e., those altered at either age) functional patterns, we used Ingenuity Pathway Analysis software to analyze genes differentially expressed between GRMD versus control dogs. As shown in Additional file 3: Table S2, several over-represented functional categories were specific to genes altered in skeletal muscle (up- or down-regulated in GRMD versus wild type dogs at either age). For instance, multiple genes important for regulation of cell death (170 genes, *p* value = 0.0001) and membrane organization (85 genes, *p* value = 0.0041) were significantly perturbed in MHG but not LV tissues in GRMD. This functional signature supports the idea that tissue-dependent gene expression alterations drive differences in disease between the two muscle types. Genes important for metabolism and energy production were likewise restricted to skeletal muscle (Additional file 3: Table S2), consistent with the notion that a metabolic phenotype contributes to dystrophic skeletal muscle disease progression.

We also performed a more comprehensive gene set enrichment analysis (GSEA) of each relevant list of differential genes (age- and tissue-matched pairwise comparisons). The most significantly enriched biological processes for genes more highly expressed in 6 month GRMD MHG relative to controls included actin filament organization inflammation (36 genes, relative enrichment score (NES) = 1.9), ECM disassembly (20 genes, NES = 1.8), inflammation (100 genes, NES = 1.6, stem cell differentiation (24 genes, NES = 1.5), muscle structure development (51 genes, NES = 15), and angiogenesis (58 genes, NES = 1.4) (Table 1). Down-regulated genes (those more highly expressed in normal dogs compared to GRMD at 6 months) were almost exclusively associated with metabolism (Table 1), including oxidation reduction (98 genes, NES = -2.6), generation of precursor metabolites and energy (45 genes, NES = -2.5), cellular respiration (24 genes, NES = -2.4), mitochondrion organization (73 genes, NES = -2.4), and glucose metabolism (21 genes, NES = -2.1). For the most part, down-regulated functional categories were more significant (lower *p* value, higher magnitude of NES) than up-regulated ontologies. Most notably, however, there was a clear lack of “functional variety” in down-regulated genes (i.e., a catabolic theme) that was not observed for genes more highly expressed in MHG of 6 month-old GRMD (Table 1).

**Table 1:** Functional analysis results of GRMD-induced gene alterations

GO Biological Process/Pathway	MHG at 6 m			LV at 6 m		
	*No*	*NES*	*p val*	*No*	*NES*	*p val*
Actin filament organization	36	1.91	0.001	-		
Regulation of extrinsic apoptotic pathway	37	1.70	0.001	-		
Regulation of cytoskeleton organization	31	1.79	0.0021	-		
ECM disassembly	20	1.78	0.0064	-		
Endothelial cell differentiation	15	1.77	0.0078	-		
Regulation of actin filament organization	57	1.64	0.0081	-		
Protein localization to organelle	55	1.58	0.006	-		
Inflammatory response	100	1.55	0.002	-		
Stem cell differentiation	24	1.54	0.0305	-		
Positive regulation of cell-substrate adhesion	24	1.54	0.0369	-		
Regulation of ion homeostasis	32	1.52	0.0327	-		
Immune system process	369	1.50	0.001	-		
Endocytosis	111	1.50	0.006	-		
Regeneration	36	1.50	0.0369	-		
Muscle structure development	51	1.46	0.0433	-		
Regulation of cell-cell adhesion	74	1.45	0.025	-		
Angiogenesis	58	1.44	0.0351	-		
Cell motility	167	1.43	0.008	-		
Regulation of cell proliferation	233	1.39	0.012	19	ns	
Regulation of cell differentiation	206	1.38	0.013	20	ns	
Cellular macromolecule localization	149	1.35	0.028	-		
Oxidation reduction	98	−2.82	0.001	16	ns	
Carboxylic acid catabolism	29	−2.58	0.001	-		
Generation of precursor metabolites and energy	45	−2.45	0.001	-		
Cellular respiration	24	−2.44	0.001	-		
Mitochondrion organization	73	−2.35	0.001	-		
Organonitrogen compound metabolism	153	−2.24	0.001	28	ns	
Coenzyme metabolism	39	−2.23	0.002	-		
Small molecule metabolism	176	−2.20	0.001	30	ns	
Catabolic process	142	−2.18	0.001	19	ns	
Hexose metabolism	22	−2.12	0.0042	-		
Glucose metabolism	21	−2.05	0.0058	-		
Regulation of ketone metabolism	26	−2.05	0.002	-		
Cofactor metabolism	46	−1.93	0.0038	-		
Macroautophagy	27	−1.93	0.0039	-		
Nitrogen compound transport	31	−1.93	0.0118	-		
Protein ubiquitination	63	−1.88	0.0062	-		
Organophosphate metabolism	84	−1.86	0.0117	-		
Small molecule biosynthesis	48	−1.85	0.0096	-		
NTP metabolism	21	−1.82	0.0081	-		
Autophagy	33	−1.79	0.022	-		
Cellular amino acid catabolism	21	−1.78	0.0135	-		
Alpha amino acid catabolism	17	−1.74	0.0259	-		
Macromolecule metabolism	60	−1.71	0.0279	-		
Anion transmembrane transport	21	−1.63	0.041			
Carbohydrate metabolism	63	−1.62	0.0299	-		
Acyl-CoA metabolism	18	−1.62	0.0433	-		
Regulation of transport	120	ns		19	−2.11	0.001
	MHG at 12 m			LV at 12 m		
	*No*	*NES*	*p val*	*No*	*NES*	*p val*
DNA metabolism	18	1.80	0.0045	-		
Lipid biosynthesis	15	1.66	0.0216	-		
Cell projection organization	37	1.62	0.0249	-		
Cellular response to stress	38	1.62	0.0227	-		
Cell proliferation	19	1.53	0.0473	-		
Regulation of cellular protein localization	22	1.53	0.0378	-		
Regulation of organelle organization	33	1.50	0.041	-		
Regulation of transport	-			19	−1.76	0.013

Functional analysis was performed using GSEA software, as detailed in the methods section. Positive and negative NES values respectively represent pathways enriched or down-regulated in GRMD animals compared to healthy counterparts. Nominal *p* values (p val) are shown, as are overlapping categories in skeletal (MHG) and left ventricular (LV) tissues for animals aged 6 months (6 m, top part of table) and 12 m (lowermost art of table). GO = gene ontology. No = number of differentially expressed genes in the category listed. NES = normalized (or relative) enrichment score. ns = not significant

Surprisingly, these enriched functions were not recapitulated in 12 month-old animals. The most significantly enriched functional categories of genes up-regulated in MHG from these older animals were DNA metabolism and lipid biosynthesis (Table 1). Significantly enriched functional categories observed in MHG of 6 month-old dogs were likewise not observed in LV at either age (Table 1). Only one biological process, regulation of transport, was significantly enriched in LV of GRMD at either age (19 genes, NES = -2.1 and -1.8 at 6 months and 12 months, respectively).

### Skeletal muscle-specific transcriptional profile: the metabolic phenotype

A direct comparison of age-matched MHG and LV tissues resulted in large lists of differentially expressed genes consistent with known differences in these two tissue types, as expected (Additional file 4: Figure S2). To eliminate those gene expression changes driven primarily by tissue specificity, we instead compared GRMD to normal for LV and MG tissues and used those separately generated lists to find GRMD driven differences between the two tissue types. To identify GRMD skeletal- and cardiac muscle-*specific* gene expression profiles, we compared the most significantly altered genes in GRMD MHG and LV at 6 months of age. For this analysis, we chose a more stringent significance cutoff (*p* value < 0.01 and 2-fold) to eliminate potentially false positive differences between GRMD-mediated changes in the two tissue types, such as *p* value = 0.049 for the MHG comparison and *p* value = 0.051 for the same comparison in LV for example). Based on these criteria, there were a total of 1,172 annotated genes that were altered in MHG from young GRMD dogs which were unchanged in matched LV samples. The majority of these (893 transcripts) were up-regulated in GRMD dogs relative to wild type controls, with only 279 more highly expressed in the wild type control animals.

Multiple pathways were associated with genes more highly expressed in 6 month GRMD MHG, including those related to inflammation (e.g., leukocyte migration, chemokine signaling, complement and coagulation cascades, phagocytosis, Toll-like receptor signaling), cytoskeletal regulation, ECM interaction/adhesion, cell cycle, platelet activation, growth and survival signaling pathways (e.g., NF-κB, p53, PI3K- Akt) and catabolic metabolism (e.g.,. lysosome, glycan degradation) (Additional file 5: Figure S3). Pathways for down-regulated genes (those more highly expressed in MHG of 6 month-old normal dogs relative to GRMD), on the other hand were almost exclusively metabolic (e.g., TCA cycle, fatty acid biosynthesis and degradation, AMPK signaling, glycolysis/gluconeogenesis, amino acid metabolism, insulin signaling) (Additional file 6: Figure S4). MHG from 12 month-old GRMD included pathways that were similar to, albeit fewer than, those found in younger dogs for both up- and down-regulated genes (Additional file 7: Figure S5).

**Fig. 2:**
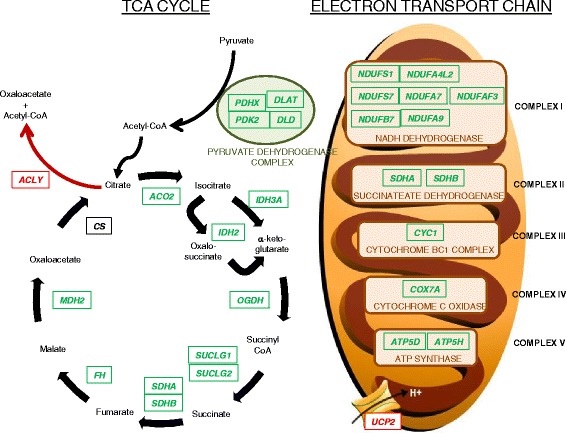
Diagram of TCA cycle and electron transport chain is shown, overlaid with gene expression results from GRMD versus wild type skeletal muscle samples. All listed genes, except for citrate synthase (*CS*), were differential between GRMD and wild type dogs, and each is colored according to direction of difference. *Green* genes represent those that were more highly expressed in wild type relative to GRMD dogs. Only two genes (*ACLY* and *UCP2*, colored in *red*) were more highly expressed in GRMD relative to control dogs

Consistent with functional and pathway analyses, manual examination of MHG-specific genes revealed a metabolic pattern consistent with a general perturbation of multiple metabolism and energy production pathways. For example, three out of four of the pyruvate dehydrogenase subunits were down-regulated, along with most of the TCA cycle enzymes and several electron transport chain components (Fig. 2). Conversely, the gene encoding ATP citrate lyase (*ACLY*), which functions counter to the other TCA cycle enzymes by synthesizing acetyl-CoA, was up-regulated, as was the transcript for the mitochondrial membrane potential de-coupler 2 (*UCP2*) that un-couples oxidative phosphorylation from ATP synthesis with energy instead dissipated as heat (Fig. 2).

**Fig. 3:**
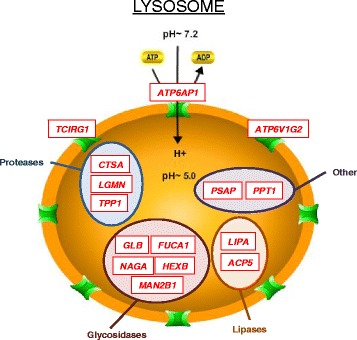
Diagram of lysosome genes that were more highly expressed in GRMD relative to wild type dogs is shown. All listed genes were more highly expressed in GRMD versus wild type skeletal muscle (red for up-regulation). Genes are grouped (*colored circles*) based on substrate type: proteases (*blue*), glycosidases (*maroon*), lipases (*dark orange/brown*), and other (*purple*). Genes encoding membrane proteins are displayed on the lysosomal periphery

**Fig. 4:**
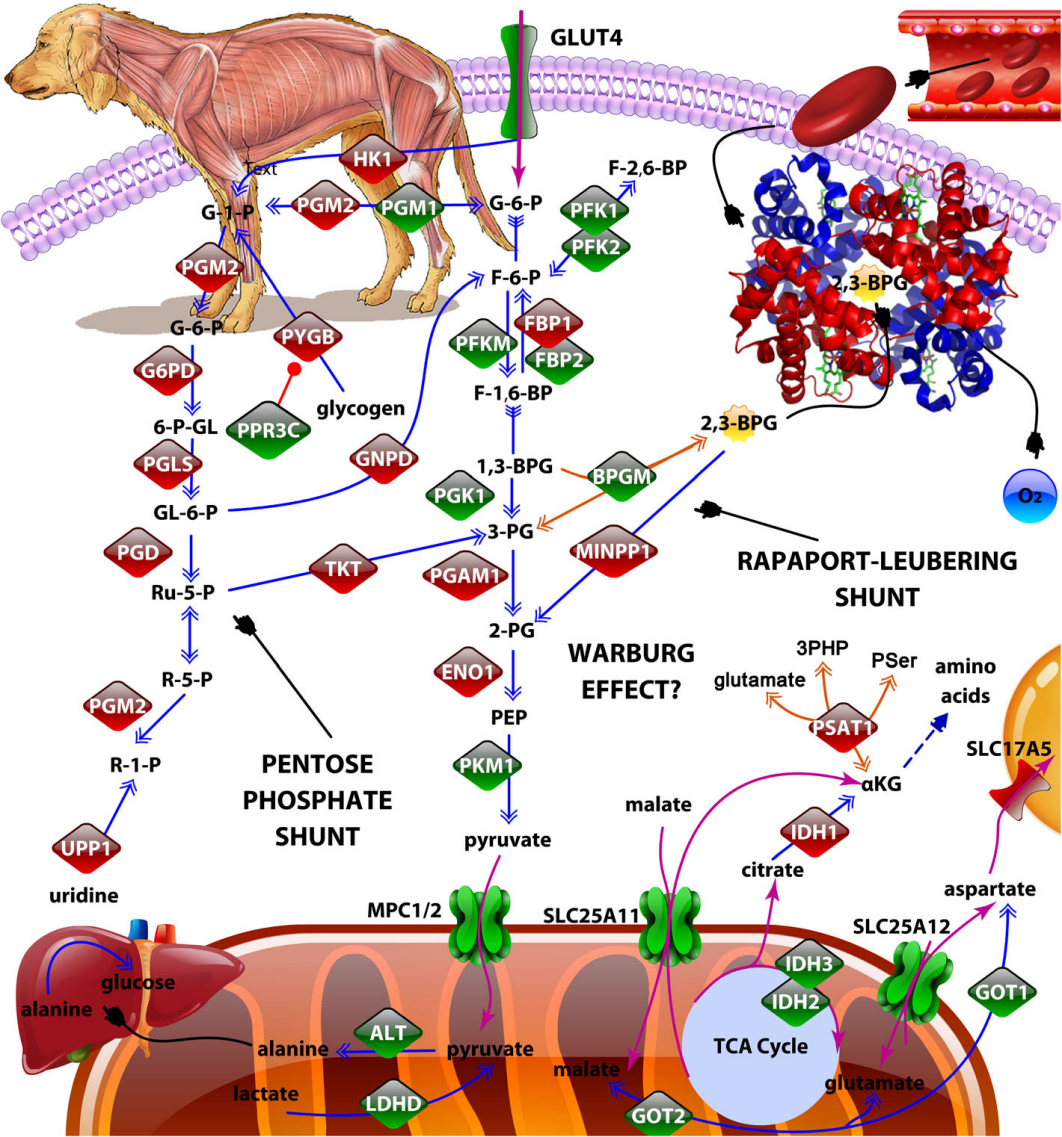
Diagram of glycolysis is shown, including enzymes whose transcripts were more highly expressed in GRMD (*red*) or wild type dogs (*green*). Metabolic transporters are also shown. The information was compiled using various public databases, and the figure was produced using Protein Lounge pathway builder software (ePath3D). The hemoglobin molecule was obtained from the Protein Databank (www.rcsb.org). The embedded dog illustration showing dystrophic musculature (*top left*) is an original work of art produced by Dave Carlson (Carlson-Art.com). Note: Common protein symbols are shown, some of which are represented by several different synonyms or have different names for their respective transcripts. For instance, MCP1 and 2 are also called BP44 and BP44L. PFK2 is encoded by *PFKFB3*

**Fig. 5:**
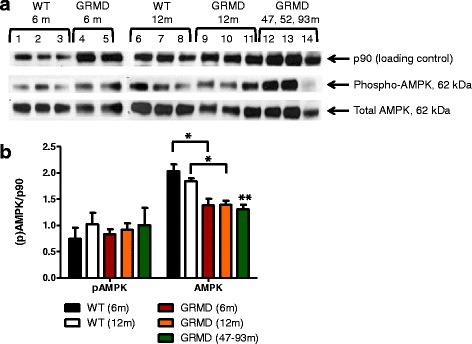
**a** Western blots for p-AMPK, total AMPK and p90 (loading control) in skeletal muscle tissues of GRMD and normal (wild type) dogs, including the three older GRMD dogs (47, 52, and 93 months-old) [17], are shown. **b** Bar graph showing semi-quantification of Western blot results for p-AMPK and AMPK, as calculated by densitometry, for skeletal muscle tissues from normal dogs aged 6 (*black* bars) or 12 (*white bars*) months, and GRMD dogs aged 6 (*red bars*), 12 (*orange* bars), or 47–93 (*green striped bars*) months. Asterisks indicate significance (*p < 0.05, **p < 0.01)

Multiple lysosome enzymes were also up-regulated in GRMD skeletal muscle (Fig. 3), possibly as a re-salvage energy source, and the glycolytic pathway and associated shunts were altered (Fig. 4) suggesting conversion to alternative energy sources instead of, or in addition to, glycolysis. Our findings were consistent with dysregulation of the AMPK pathway, which when activated in muscle contributes to a slower, more oxidative phenotype. Indeed, the gene encoding the catalytic subunit of AMPK (*PRKAA2*, *p* value 3.7 × 10^−6^, -2.3-fold) was down-regulated in GRMD MHG, as was the gene encoding the important downstream effector, PGC-1α (*PPARGC1A*, *p* value 9.6 × 10^−3^, -2.0-fold). We confirmed down-regulation of AMPK in GRMD skeletal muscle by Western blot analysis (Fig. 5) but did not find any difference in the phosphorylation status of AMPK in these same tissues. The AMPK levels in GRMD dogs were significantly lower than those from respective normal controls. AMPK levels were lower in the older (47–93 months) versus younger GRMD dogs, although this difference was not significant. Surprisingly, protein levels of AMPK were undetectable in matched cardiac tissue from these same animals (data not shown).

Interestingly, *GLUT4*, which encodes an insulin-regulated facilitative glucose transporter, was also significantly down-regulated in MHG from 6 month-old dogs. This is likely an important finding, given the previously suggested role of *GLUT4* alterations in insulin resistance in DMD patients [30].

### Cardiac muscle-specific transcriptional profile: the peroxisomal bypass

**Fig. 6:**
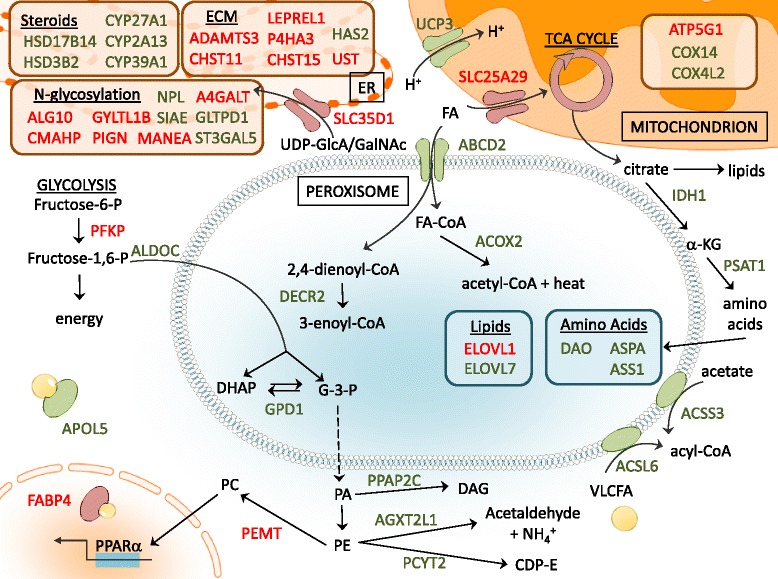
Diagram of peroxisomal beta-oxidation and other metabolic changes observed in GRMD LV tissues is shown. Proteins shown represent up-regulation (*red*) or down-regulation (*green*) of associated transcripts in dystrophic dogs as compared to normal controls

**Fig. 7:**
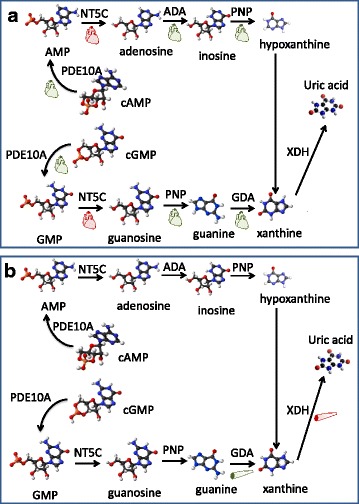
Diagram of nucleic acid gene expression differences in GRMD versus wild type dogs is shown. Panel **a**, Small red heart icons are shown below the names of enzymes whose transcripts were more highly expressed in LV of GRMD compared to wild type dogs. Green heart icons accompany those encoded by genes that were more highly expressed in wild type versus GRMD dogs. Panel **b**, Small skeletal muscle icons are shown below the names of enzymes whose transcripts were more highly expressed in MHG of GRMD compared to wild type dogs (*red*) or in wild type versus GRMD dogs (*green*)

In contrast to skeletal muscle findings, the expression levels of the genes encoding AMPK and PGC-1α were unaltered in LV tissues from 6 and 12-month-old GRMD dogs when compared to age-matched controls, despite alteration in 5 genes associated with the AMPK signaling pathway in the older animals (Additional file 8: Figure S6). Similarly, mass down-regulation of critical energy production genes was not observed for LV tissue, nor was there up-regulation of multiple lysosomal genes. *En bloc* down-regulation of genes involved in peroxisome-specific beta-oxidation and uric acid production was, instead, seen in parallel with the up-regulation of key entry points for mitochondrial-based energy production (Fig. 6). Interestingly, the *NT5C* gene encoding cytosolic 5′,3′-pyrimidine nucleotidase (also called deoxy-5′-nucleotidase, or dNT-1) was up-regulated in GRMD versus wild type dogs, concomitant with down-regulation of genes encoding enzymes that function up-stream and downstream of *NT5C* (Fig. 7). While interpretation of these enzymatic changes would require further experimentation, two possible consequences might include accumulation of adenosine in the dystrophic heart (but not skeletal muscle) and up-regulation of uric acid production in dystrophic skeletal muscle (but not in the heart).

## Discussion

Duchenne muscular dystrophy is characterized by striking temporal differential involvement of cardiac and skeletal muscles [31], despite shared dystrophin protein deficiency and similar physiological functions in the two muscle types. The GRMD model provides an excellent platform for studying human DMD, largely because of the remarkable level of similarity between dogs and humans in general (e.g., in body structure) as well as likenesses in DMD/GRMD disease features [16]. In this study, we endeavored to discern whether the distinct cardiac and skeletal phenotypes in GRMD (and, by extension, DMD) are associated with differential gene expression within the respective muscle groups. By evaluating distinct ages representing different stages of GRMD disease, and two different tissue types historically observed to show contrasting levels of disease over this period, we were able to compare expression profiles in context with disease state [19].

The global transcriptome of MHG but not LV from 6 month-old GRMD dogs was profoundly altered. However, consistent with subsequent relative stabilization of skeletal muscle disease, these early expression changes were not sustained at 12 months. Looking more specifically at metabolic pathways in MHG from 6-month-old GRMD dogs, we identified a gene expression signature consistent with a “metabolic crisis” in DMD studies [9]. This canonical expression signature was entirely absent from matched GRMD LV tissues. Thus, our findings suggest differential metabolic dysfunction may contribute to distinct pathological phenotypes in these two muscle types. The most obvious transcriptional alterations that could contribute to metabolic dysfunction were down-regulation of dozens of energy production-associated molecules, most notably all of the TCA cycle enzymes and multiple electron transport components. Likewise, the majority of glycolytic pathway-associated enzymes were altered in a manner consistent with shunting of glycolytic molecule intermediates into alternative pathways, such as the anabolic pentose phosphate pathway. This is consistent with generalized muscle atrophy of any cause [11] and DMD in particular [29].

Gene expression alterations in MHG muscle from GRMD dogs also suggested that the Rappaport-Leubering Shunt was interrupted. For example, there was down-regulation of the gene encoding bisphosphoglycerate mutase (*BPGM*), the enzyme that produces 2,3 bisphosphoglycerate (2,3-BPG), an allosteric effector which enhances release of oxygen from hemoglobin to the tissues. Multiple inositol-polyphosphate phosphatase 1 (*MINP1*), whose product converts 2,3-BPG to 2-phosphoglycerate, was instead up-regulated. Decrease in 2,3-BPG levels could lead to impaired tissue oxygenation, such as that observed in iron deficient anemia or chronic respiratory disease with hypoxia. Consistent with this hypothesis, we also observed a small but significant reduction (1.4-fold, *p* value = 0.006) in the myoglobin gene in MHG (but not LV) tissues of GRMD animals.

## Conclusions

When considered together, these various gene expression alterations provide a plausible mechanism for metabolic changes observed in the clinical setting. Lowered glucose uptake and glycolytic changes may contribute to insulin resistance. Impaired tissue oxygenation, along with a reduction in TCA and electron transport energy production, would support a shift from aerobic to anaerobic respiration, causing in essence a Warburg effect. In concert, these metabolic events, paired with the high-energy demands of regenerating muscle, almost certainly contribute to disease progression. In summary, this study provides novel insight into the metabolic gene expression profile of dystrophic cardiac tissue, which is unique from matched skeletal muscle of GRMD dogs. Further, these findings suggest a plausible mechanistic explanation for the chronological pattern of disease in these two different muscle types.

## Additional files

Additional file 1: Table S1. Statistically significant gene list. Column A = Affymetrix unique probe set ID, Column B = Gene ID, Column C = gene symbol, Column D = RefSeq ID, Column E = gene assignment, Column F = gene title, Column G = gene symbol, Column H = ANOVA-generated *p* values for all groups, Column I = ANOVA-generated *p* values for comparison of left ventricular (LV) tissues from 6 month-old (6 m) Golden Retriever Muscular Dystrophy (GRMD) dogs versus LV from 6 m normal controls, Column J = Fold difference for [LV 6 m GRMD/LV 6 m normal], Column K = ANOVA-generated *p* values for comparison of LV 12 m GRMD versus LV 12 m normal, Column L = Fold difference for [LV 12 m GRMD/LV 12 m normal], Column M = ANOVA-generated *p* values for comparison of medial head of the gastrocnemius (MG) from 6 m GRMD versus MG 6 m normal, Column *N* = Fold difference for [MG 6 m GRMD/MG 6 m normal], Column O = ANOVA-generated *p* values for comparison of MG 12 m GRMD versus MG 12 m normal, Column *P* = Fold difference for [MG 12 m GRMD/MG 12 m normal]. Gene annotations were obtained within Partek (Columns B-C) and using Affymetrix NetAffx database information (Columns D-G). (XLSX 995 kb)

Additional file 2: Figure S1. Hierarchical clustering of 5,835 significantly altered transcripts is shown. Rows represent individual samples, labeled according to group, and columns represent individual transcripts. Bright red, bright blue and gray represent highest, lowest and median normalized and scaled signal intensity values, respectively. (DOCX 116 kb)

Additional file 3: Table S2. Functional analysis results of GRMD-induced gene alterations. MHG = medial head of the gastrocnemius, LV = left ventricle, B-H *p* val = Benjamini & Hochberg-corrected *p* value, No. = number of differentially expressed genes in the given functional category, ns = not significant, Dash (“-”) indicates not identified in the analysis. (DOCX 16 kb)

Additional file 4: Figure S2. Volcano plot is shown highlighting gene expression differences between dystrophic left ventricle (LV) and medial head of the gastrocnemius (MHG) skeletal muscle of golden retriever muscular dystrophy (GRMD) dogs at 6 months. The y-axis displays *p* values, and fold-differences are plotted on the x-axis for comparison of GRMD LV versus MHG (LV/MHG). Each circle represents an individual probe set (gene) and is colored based on fold-difference (blue for genes with lower expression in GRMD MHG and higher expression in LV, red for genes with higher expression values in LV and lower in MHG, and gray for no change), as shown in the legend. Orange lines mark cut-off values (*p* value < 0.05, fold-difference > 2). Official gene symbols are indicated to highlight major expression differences between LV and MHG. (PPTX 136 kb)

Additional file 5: Figure S3. Plot shows over-represented pathways for genes up-regulated in 6 m GRMD MHG. Statistical significance (smaller *p* values = larger numbers = greater significance) is shown on the x-axis, and numbers of genes altered per pathway is shown to the right of each bar. (DOCX 32 kb)

Additional file 6: Figure S4. Plot shows over-represented pathways for genes down-regulated in 6 m GRMD MHG. Statistical significance (smaller *p* values = larger numbers = greater significance) is shown on the x-axis, and numbers of genes altered per pathway is shown to the right of each bar. (DOCX 35 kb)

Additional file 7: Figure S5. Plot shows over-represented pathways for genes up- (red bars) or down-regulated (green bars) in 12 m GRMD MHG. Statistical significance (smaller *p* values = larger numbers = greater significance) is shown on the x-axis, and numbers of genes altered per pathway is shown to the right of each bar. (DOCX 30 kb)

Additional file 8: Figure S6. Plot shows over-represented pathways for genes altered in 6 m (light blue bars, top) or 12 m (dark blue bars, bottom) GRMD LV. Statistical significance (smaller *p* values = larger numbers = greater significance) is shown on the x-axis, and numbers of genes altered per pathway is shown to the right of each bar. AMPK signaling pathway genes are shown below the corresponding pathway bar, colored based on directionality (red for up-regulation and green for down-regulation, respectively in GRMD versus normal dogs). (DOCX 26 kb)

## Abbreviations

2,3-BPG: 2,3 bisphosphoglycerate
ACLY: ATP citrate lyase
AMPK: 5′ AMP-activated protein kinase
ANOVA: Analysis of Variance
BDNF: Brain-derived neurotropic factor
B-H: Benjamini and Hochberg
BPGM: bisphosphoglycerate mutase
CLCN1: Chloride Voltage-Gated Channel 1
DMD: Dystrophin Muscular Dystrophy
GRMD: Golden Retriever Muscular Dystrophy
LV: Left ventricle
*mdx*: X chromosome-linked muscular dystrophy (in the mouse)
MHG: Medial head of gastrocnemius
MINP1: Multiple inositol-polyphosphate phosphatase 1
MSTN: Myostatin
MYBPC2: Myosin Binding Protein C, fast type
MYLK2: Myosin Light Chain Kinase 2
NT5C: 5′, 3′-Nucleotidase, cytosolic
OPN: Osteopontin
PGC-1α/PPARGC1A: Peroxisome Proliferator-activated Receptor-γ Coactivator
POSTN: Periostin
PRKAA2: Protein Kinase AMP-Activated Catalytic Subunit Alpha 2
SEM: Standard Error of the Mean
SNTB1: Syntrophin beta 1
TCA: Tricarboxylic Acid (citric acid cycle, Krebs cycle)
TIMP1: Tissue Inhibitor Of Metalloproteinases 1
UCP2: Uncoupling Protein 2
