# Supplementary figures and images for: GRMD cardiac and skeletal muscle metabolism gene profiles are distinct
Source: BMC Med Genomics. 2017 Apr 8;10:21. doi: 10.1186/s12920-017-0257-2 (PMC5385041; doi:10.1186/s12920-017-0257-2)

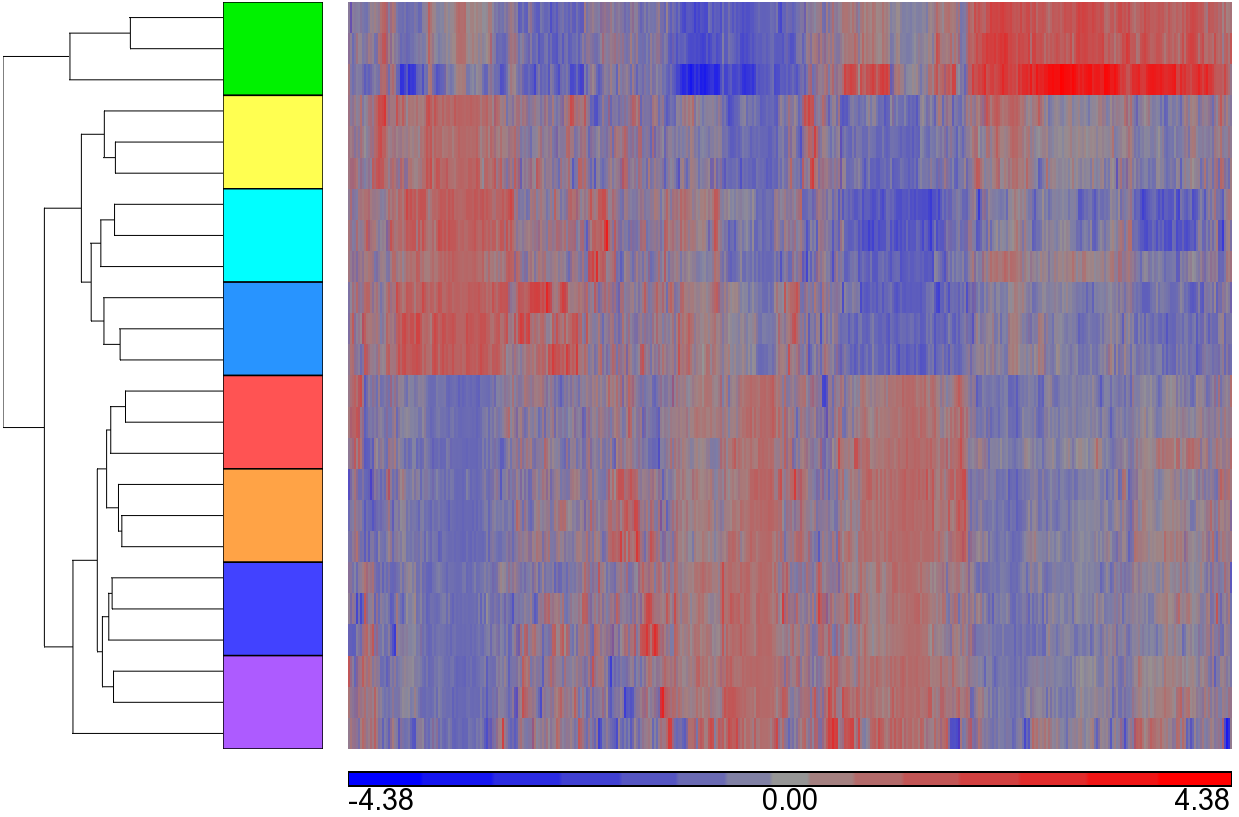


Normal 6m LV

Normal 12m LV

GRMD 6m LV

GRMD 12m LV

Normal 12m MG

Normal 6m MG

GRMD 12m MG

GRMD 6m MG

Supplement: Supplementary file 2 — Hierarchical clustering of 5,835 significantly altered transcripts is shown. Rows represent individual samples, labeled according to group, and columns represent individual transcripts. Bright red, bright blue and gray represent highest, lowest and median normalized and scaled signal intensity values, respectively. (DOCX 116 kb) [file 12920_2017_257_MOESM2_ESM.docx]

## Slide 1
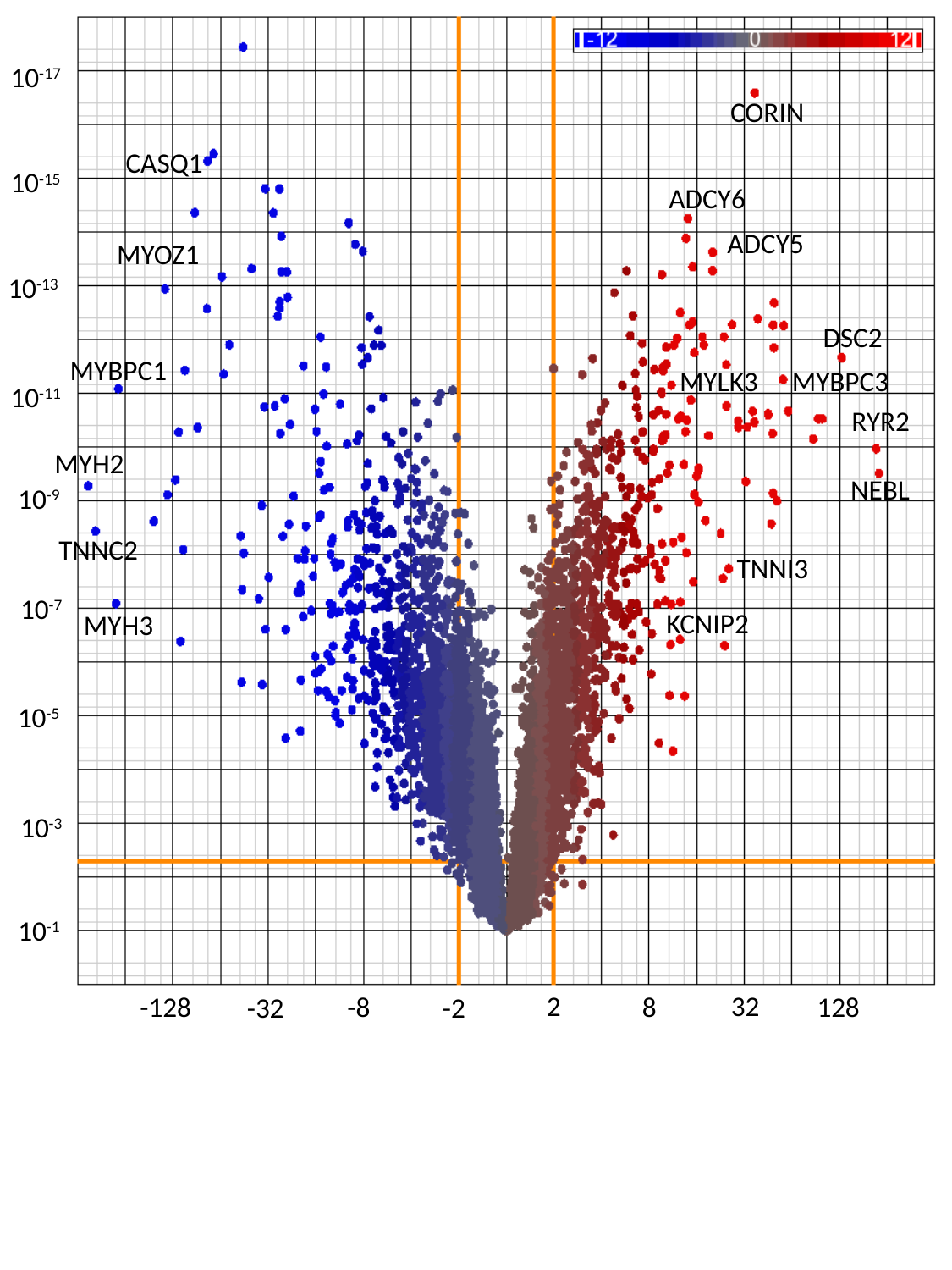

10-17
CORIN
CASQ1
10-15
ADCY6
ADCY5
MYOZ1
10-13
DSC2
MYBPC1
MYLK3
MYBPC3
10-11
RYR2
MYH2
NEBL
10-9
TNNC2
TNNI3
10-7
KCNIP2
MYH3
10-5
10-3
10-1
2
32
8
128
-128
-8
-32
-2

Supplement: Supplementary file 4 — Volcano plot is shown highlighting gene expression differences between dystrophic left ventricle (LV) and medial head of the gastrocnemius (MHG) skeletal muscle of golden retriever muscular dystrophy (GRMD) dogs at 6 months. The y-axis displays p values, and fold-differences are plotted on the x-axis for comparison of GRMD LV versus MHG (LV/MHG). Each circle represents an individual probe set (gene) and is colored based on fold-difference (blue for genes with lower expression in GRMD MHG and higher expression in LV, red for genes with higher expression values in LV and lower in MHG, and gray for no change), as shown in the legend. Orange lines mark cut-off values (p value < 0.05, fold-difference > 2). Official gene symbols are indicated to highlight major expression differences between LV and MHG. (PPTX 136 kb) [file 12920_2017_257_MOESM4_ESM.pptx]
