# Supplementary material for: GRMD cardiac and skeletal muscle metabolism gene profiles are distinct
Source: BMC Med Genomics. 2017 Apr 8;10:21. doi: 10.1186/s12920-017-0257-2 (PMC5385041; doi:10.1186/s12920-017-0257-2)
Supplement: Supplementary file 3 — Functional analysis results of GRMD-induced gene alterations. MHG = medial head of the gastrocnemius, LV = left ventricle, B-H p val = Benjamini & Hochberg-corrected p value, No. = number of differentially expressed genes in the given functional category, ns = not significant, Dash (“-”) indicates not identified in the analysis. (DOCX 16 kb) [file 12920_2017_257_MOESM3_ESM.docx]

| **Function** | **MHG** | | **LV** | |
| --- | --- | --- | --- | --- |
|  | B-H p val | No. | B-H p val | No. |
| Regulation of cell proliferation | 1.01 X 10^-05^ | 173 | ns | 23 |
| Cofactor Metabolism | 1.54 X 10^-05^ | 61 | - | - |
| Vasculature development | 2.61 X 10^-05^ | 71 | 8.76 X 10^-04^ | 26 |
| Response to wounding | 2.72 X 10^-05^ | 124 | ns | 11 |
| Cell motion | 3.83 X 10^-05^ | 113 | ns | 16 |
| Blood vessel development | 6.01 X 10^-05^ | 68 | 6.77 X 10^-04^ | 25 |
| Regulation of cell death/apoptosis | 1.25 X 10^-04^ | 170 | - | - |
| Angiogenesis | 3.54 X 10^-04^ | 45 | 1.00 X 10^-02^ | 17 |
| Muscle organ development | 6.38 X 10^-04^ | 57 | ns | 13 |
| Blood vessel morphogenesis | 6.38 X 10^-04^ | 57 | 1.01 X 10^-03^ | 23 |
| Acetyl-CoA metabolism/TCA cycle | 1.31 X 10^-03^ | 16 | - | - |
| Membrane organization | 4.07 X 10^-03^ | 85 | - | - |
| Cell adhesion | 5.40 X 10^-03^ | 139 | 1.80 X 10^-02^ | 43 |
| Striated muscle cell differentiation | 6.60 X 10^-03^ | 28 | - | - |
| Intracellular transport | 6.87 X 10^-03^ | 131 | - | - |
| Actin filament-based process | 7.08 X 10^-03^ | 58 | - | - |
| Monosaccharide metabolism | 8.28 X 10^-03^ | 54 | - | - |
| Tube development | 2.77 X 10^-02^ | 51 | 1.00 X 10^-02^ | 21 |
| Response to oxygen levels | 1.12 X 10^-02^ | 38 | ns | 11 |
| Response to cytokines | 1.45 X 10^-02^ | 25 | ns | 8 |
| Biological adhesion | 5.69 X 10^-03^ | 139 | 1.52 X 10^-02^ | 43 |
| Oxidation of organic compounds for energy | 1.59 X 10^-02^ | 38 | ns | 9 |
| Leukocyte activation/immune response | 1.63 X 10^-02^ | 15 | ns | 10 |
| Striated muscle tissue development | 1.64 X 10^-02^ | 33 | - | - |
| Hexose metabolism | 1.67 X 10^-02^ | 47 | ns | 4 |
| Cellular respiration | 2.24 X 10^-02^ | 28 | - | - |
| Blood coagulation | 2.24 X 10^-02^ | 29 | - | - |
| Positive regulation of cell differentiation | 2.38 X 10^-02^ | 53 | ns | 15 |
| Cell projection assembly | 2.41 X 10^-02^ | 25 | ns | 22 |
| Cytoskeletal organization | 2.61 X 10^-02^ | 89 | - | - |
| Response to hypoxia | 2.69 X 10^-02^ | 35 | - | 10 |
| ECM organization | 2.87 X 10^-02^ | 29 | - | - |
| Muscle cell differentiation | 3.55 X 10^-02^ | 32 | - | - |
| Vitamin transport | 4.23 X 10^-02^ | 11 | - | - |
