# Supplementary material for: GRMD cardiac and skeletal muscle metabolism gene profiles are distinct
Source: BMC Med Genomics. 2017 Apr 8;10:21. doi: 10.1186/s12920-017-0257-2 (PMC5385041; doi:10.1186/s12920-017-0257-2)
Supplement: Supplementary file 4 [file 12920_2017_257_MOESM5_ESM.docx]

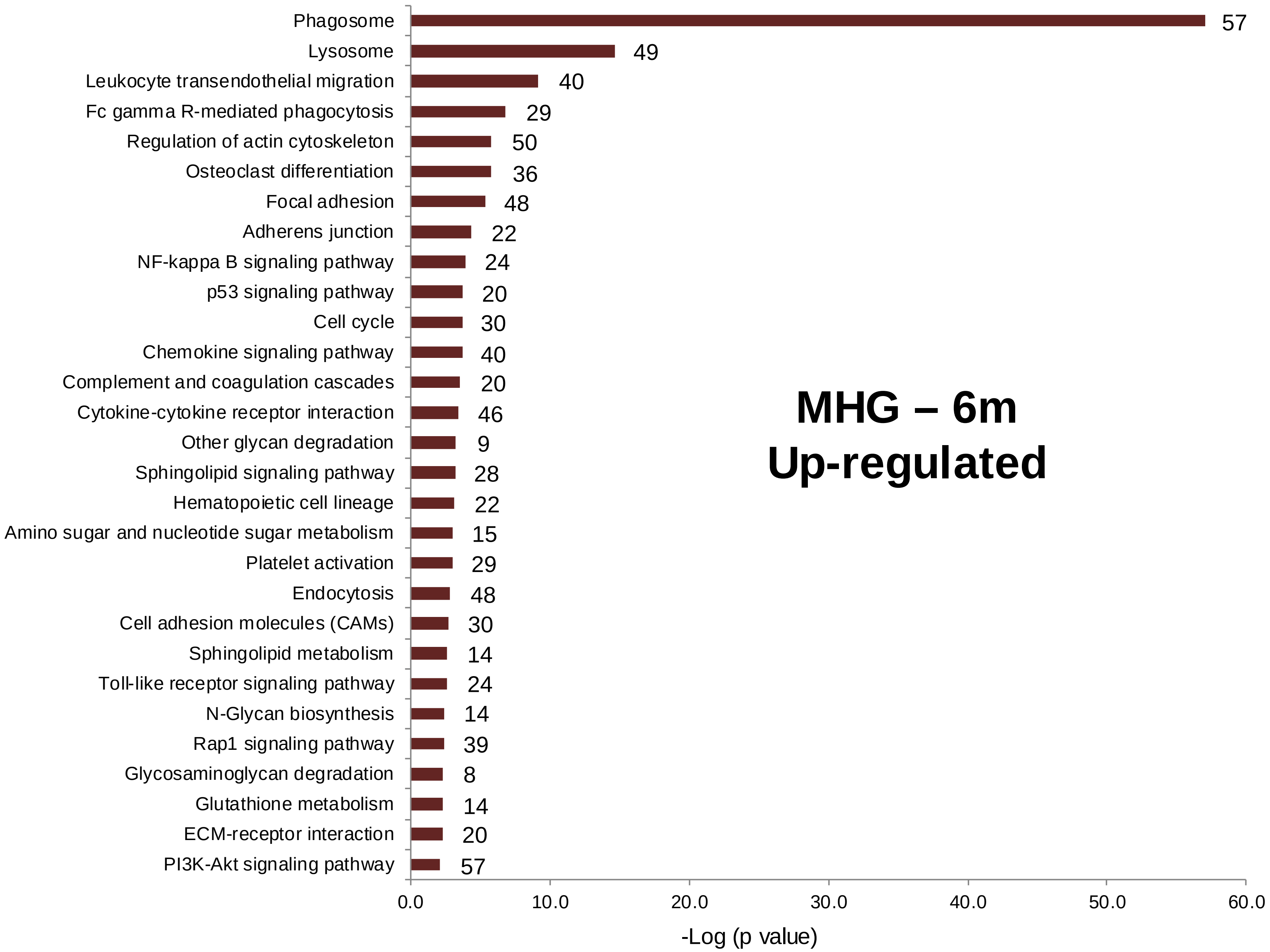
MHG – 6m
Up-regulated
Phagosome 57
Lysosome 49
Leukocyte transendothelial migration 40
Fc gamma R-mediated phagocytosis 29
Regulation of actin cytoskeleton 50
Osteoclast differentiation 36
Focal adhesion 48
Adherens junction 22
NF-kappa B signaling pathway 24
p53 signaling pathway 20
Cell cycle 30
Chemokine signaling pathway 40
Complement and coagulation cascades 20
Cytokine-cytokine receptor interaction 46
Other glycan degradation 9
Sphingolipid signaling pathway 28
Hematopoietic cell lineage 22
Amino sugar and nucleotide sugar metabolism 15
Platelet activation 29
Endocytosis 48
Cell adhesion molecules (CAMs) 30
Sphingolipid metabolism 14
Toll-like receptor signaling pathway 24
N-Glycan biosynthesis 14
Rap1 signaling pathway 39
Glycosaminoglycan degradation 8
Glutathione metabolism 14
ECM-receptor interaction 20
PI3K-Akt signaling pathway 57
0.0
10.0
20.0
30.0
40.0
50.0
60.0
-Log (p value)
