# Supplementary material for: GRMD cardiac and skeletal muscle metabolism gene profiles are distinct
Source: BMC Med Genomics. 2017 Apr 8;10:21. doi: 10.1186/s12920-017-0257-2 (PMC5385041; doi:10.1186/s12920-017-0257-2)
Supplement: Supplementary file 5 [file 12920_2017_257_MOESM6_ESM.docx]

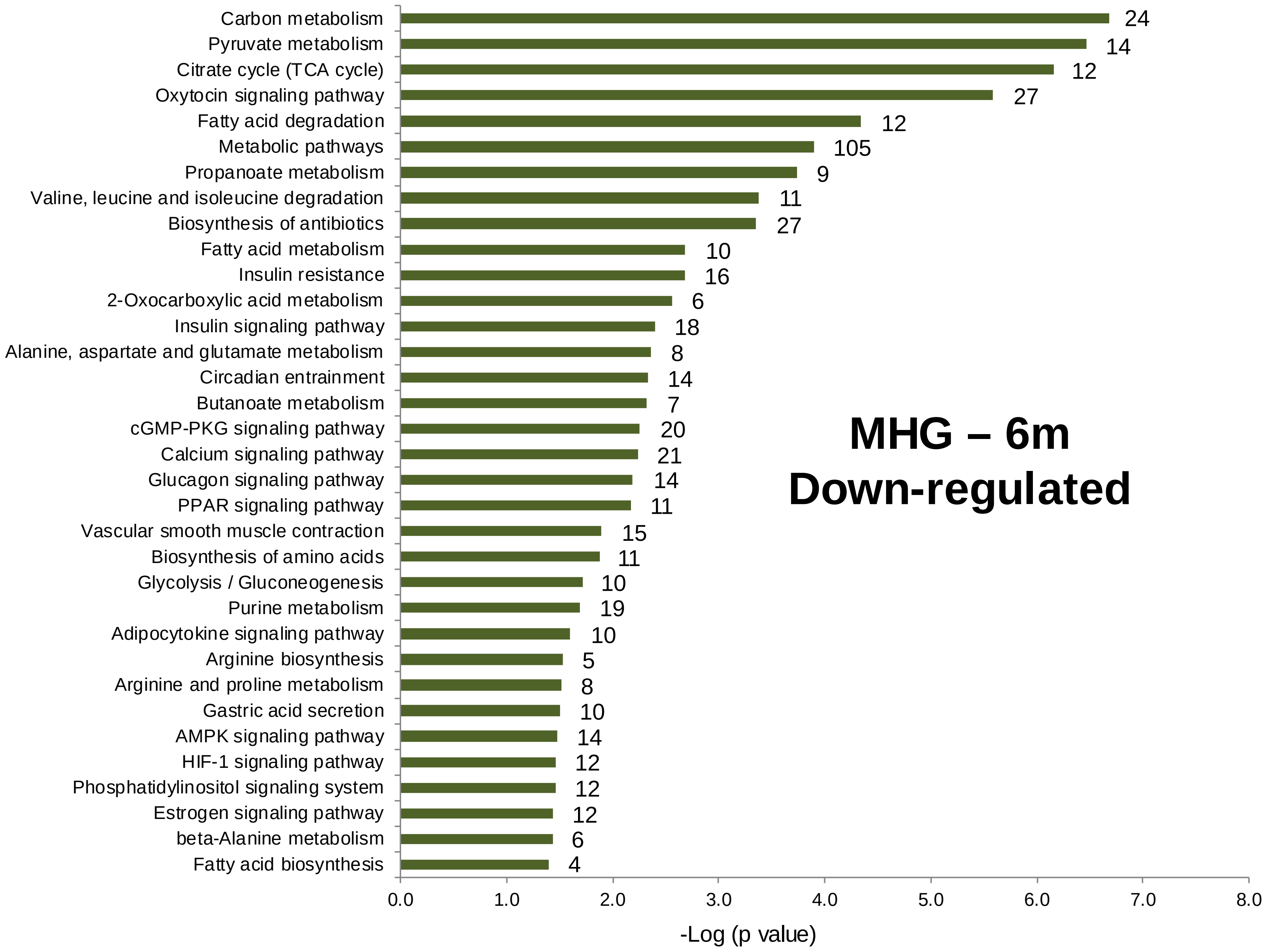

Carbon metabolism
Pyruvate metabolism
Citrate cycle (TCA cycle)
Oxytocin signaling pathway
Fatty acid degradation
Metabolic pathways
Propanoate metabolism
Valine, leucine and isoleucine degradation
Biosynthesis of antibiotics
Fatty acid metabolism
Insulin resistance
2-Oxocarboxylic acid metabolism
Insulin signaling pathway
Alanine, aspartate and glutamate metabolism
Circadian entrainment
Butanoate metabolism
cGMP-PKG signaling pathway
Calcium signaling pathway
Glucagon signaling pathway
PPAR signaling pathway
Vascular smooth muscle contraction
Biosynthesis of amino acids
Glycolysis / Gluconeogenesis
Purine metabolism
Adipocytokine signaling pathway
Arginine biosynthesis
Arginine and proline metabolism
Gastric acid secretion
AMPK signaling pathway
HIF-1 signaling pathway
Phosphatidylinositol signaling system
Estrogen signaling pathway
beta-Alanine metabolism
Fatty acid biosynthesis
24
14
12
27
12
105
9
11
27
10
16
6
18
8
14
7
20
21
14
11
15
11
10
19
10
5
8
10
14
12
12
12
6
4
MHG – 6m
Down-regulated
0.0
1.0
2.0
3.0
4.0
5.0
6.0
7.0
8.0
-Log (p value)
