# Supplementary material for: GRMD cardiac and skeletal muscle metabolism gene profiles are distinct
Source: BMC Med Genomics. 2017 Apr 8;10:21. doi: 10.1186/s12920-017-0257-2 (PMC5385041; doi:10.1186/s12920-017-0257-2)
Supplement: Supplementary file 6 [file 12920_2017_257_MOESM7_ESM.docx]

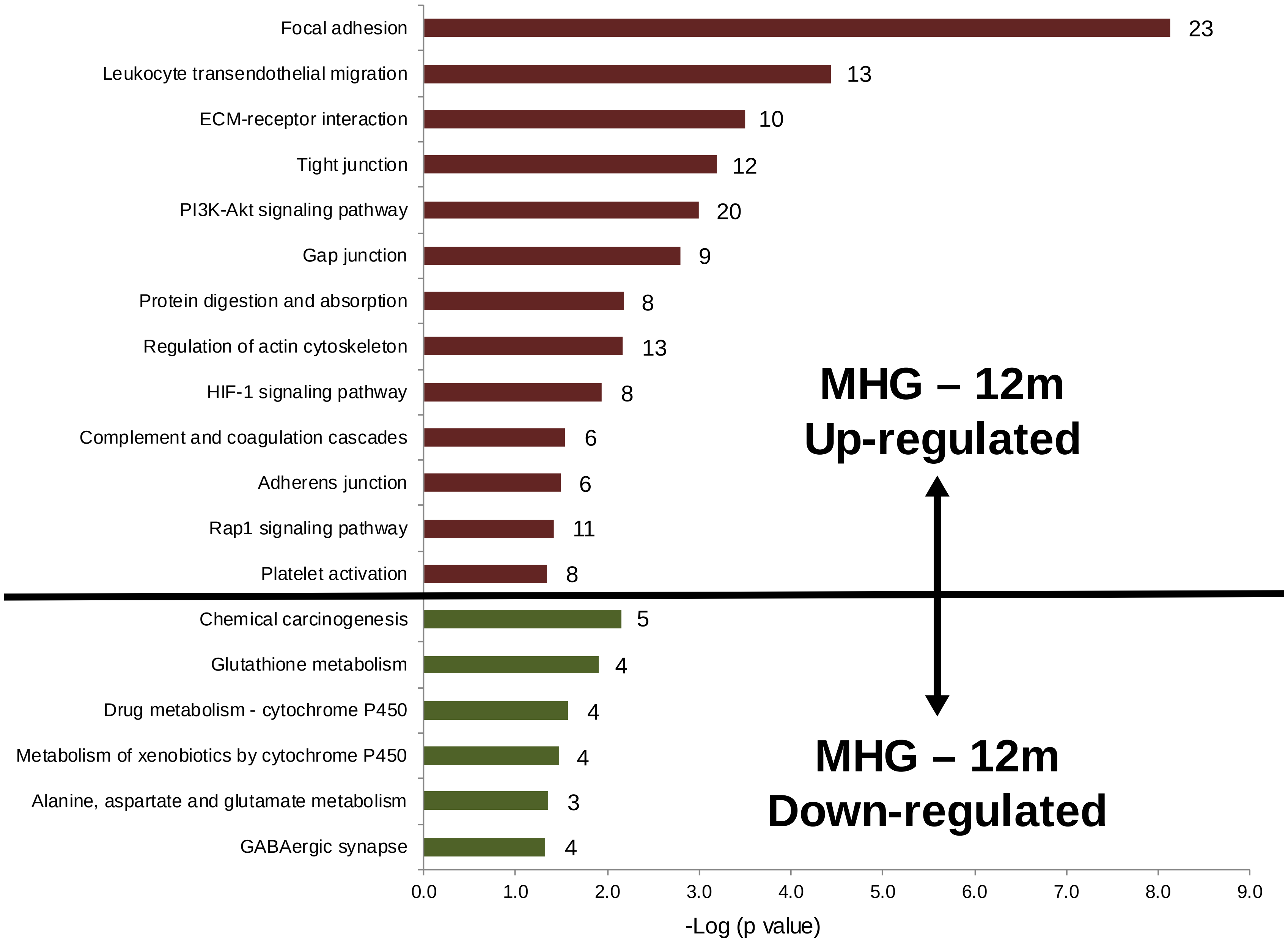
Focal adhesion
23
Leukocyte transendothelial migration
13
ECM-receptor interaction
10
Tight junction
12
PI3K-Akt signaling pathway
20
Gap junction
9
Protein digestion and absorption
8
Regulation of actin cytoskeleton
13
HIF-1 signaling pathway
8
Complement and coagulation cascades
6
Adherens junction
6
Rap1 signaling pathway
11
Platelet activation
8
Chemical carcinogenesis
5
Glutathione metabolism
4
Drug metabolism - cytochrome P450
4
Metabolism of xenobiotics by cytochrome P450
4
Alanine, aspartate and glutamate metabolism
3
GABAergic synapse
4
MHG – 12m
Up-regulated
MHG – 12m
Down-regulated
0.0
1.0
2.0
3.0
4.0
5.0
6.0
7.0
8.0
9.0
-Log (p value)
