# Supplementary material for: GRMD cardiac and skeletal muscle metabolism gene profiles are distinct
Source: BMC Med Genomics. 2017 Apr 8;10:21. doi: 10.1186/s12920-017-0257-2 (PMC5385041; doi:10.1186/s12920-017-0257-2)
Supplement: Supplementary file 7 [file 12920_2017_257_MOESM8_ESM.docx]

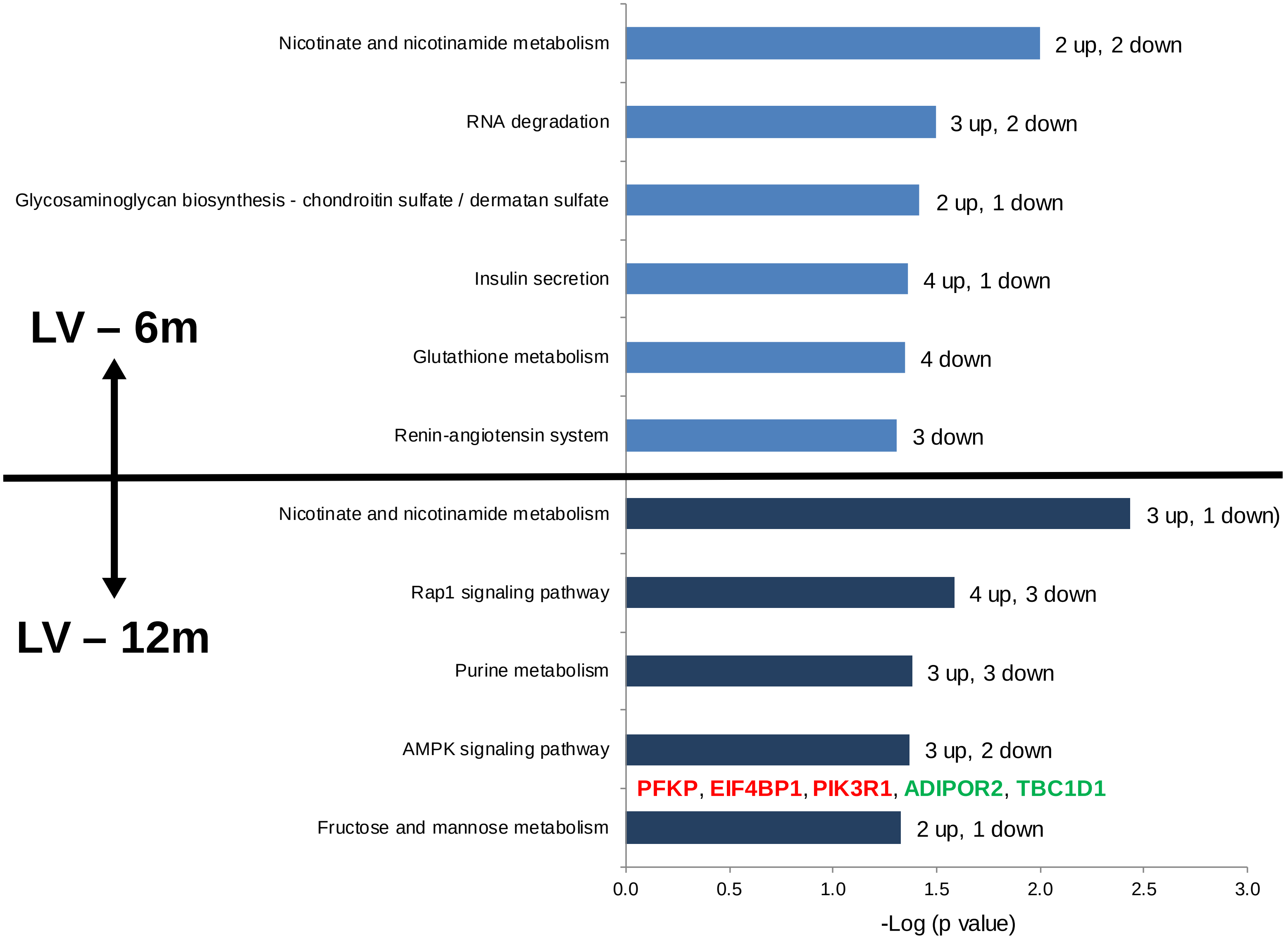
LV – 6m
LV – 12m
Nicotinate and nicotinamide metabolism
2 up, 2 down
RNA degradation
3 up, 2 down
Glycosaminoglycan biosynthesis - chondroitin sulfate / dermatan sulfate
2 up, 1 down
Insulin secretion
4 up, 1 down
Glutathione metabolism
4 down
Renin-angiotensin system
3 down
Nicotinate and nicotinamide metabolism
3 up, 1 down)
Rap1 signaling pathway
4 up, 3 down
Purine metabolism
3 up, 3 down
AMPK signaling pathway
3 up, 2 down
PFKP, EIF4BP1, PIK3R1, ADIPOR2, TBC1D1
Fructose and mannose metabolism
2 up, 1 down
0.0
0.5
1.0
1.5
2.0
2.5
3.0
-Log (p value)
